# Commentary: Opium Alkaloids in Harvested and Thermally Processed Poppy Seeds

**DOI:** 10.3389/fchem.2020.622488

**Published:** 2021-01-20

**Authors:** Daria Kleinmeier, Emily Pettengill, Benjamin W. Redan

**Affiliations:** U.S. Food and Drug Administration, Center for Food Safety and Applied Nutrition, Office of Food Safety, Bedford Park, IL, United States

**Keywords:** poppy seeds, alkaloids, thermal processing, LC-MS, morphine, codeine, thebaine

“In the article by Carlin et al. (2020), “Opium Alkaloids in Harvested and Thermally Processed Poppy Seeds,” the authors report the results of their work on the effects of thermal processing on concentrations of opium alkaloids in poppy seeds. We recognize the value of the article’s contribution to this field; however, we have several concerns with some of the author’s claims and the experimental design used to support their conclusions.

Most importantly, while the authors maintained that thermal processing under the described experimental conditions resulted in a reduction of opium alkaloids in poppy seeds, we believe that it is difficult to use their results to support such claims. In their study, the authors performed an experiment where poppy seeds were applied to the surface of bread roll dough, and the dough was baked at 190°C for 25 min. Table 8 in their manuscript does generally show higher mean values for opium alkaloids in unprocessed seeds compared to those either subjected to dry heat or applied to the surface of bread dough and exposed to the baking treatment. Yet, as noted by the authors, there was a large range in the levels of opium alkaloids in the unprocessed poppy seeds (for example, morphine in sample #1 ranged from ∼8–1,900 ng/g). The high variability in opium alkaloid concentrations makes it difficult to ascertain how the thermal processing affected levels of these compounds. High natural variation of opium alkaloid concentrations in poppy seeds has been previously reported, which has led some authors to suggest using a relatively large sample size for analysis—such as 10 g poppy seeds or greater—to reduce analyte variance (López et al., 2018). As Carlin et al. only used 200 mg of ground seeds for their analysis, this perhaps is one factor that may explain the large variability in data between replicates. Lack of reported statistical tests and relatively low number of replicates prevents clear conclusions from being drawn from these baking experiments and thus leaves room open for further experiments to address these challenges.

In addition to the high sample variability in opium alkaloid levels in poppy seeds used in the study, there are several other important aspects of the experimental design that we believe make it difficult to draw definitive conclusions from the thermal processing experiments. Because Carlin et al. analyzed seeds scraped from the top of the finished baked product, it is plausible that the reported loss of alkaloids might be due to transfer of alkaloids from the surface of the seeds to the batter. Opium alkaloids in poppy seeds can be significantly reduced by washing the surface of the seeds, but lack of reported recovery experiments by the authors leave unanswered whether losses are “true losses” due to degradation of the alkaloid compounds, or are an artifact of the compounds simply transferring to the batter (CONTAM Panel et al., 2018; Powers et al., 2018; Shetge et al., 2020). We hypothesize that follow-up experiments taking into account recovery may find higher alkaloid concentrations in the baked product than initially reported.

Moreover, although the baking experiments exposed the poppy seed-coated bread dough in an oven set at 190°C, Carlin et al. did not monitor the actual temperature of the surface of the baking dough. Due to moisture retained in dough throughout the baking process, it is important to monitor the temperature of the baked product, as there can be large differences between the set oven temperature and the surface/interior of the baked product. In contrast to the conclusions made by Carlin et al., our group’s research has found that baking poppy seeds either incorporated in a baked product (a muffin) or applied to the product’s surface resulted in no significant effect on opium alkaloid levels (See Figure 1) (Shetge et al., 2020). The results from these baking experiments were supported by determining the activation energy (*E*_a_) values and half-lives of the major opium alkaloids in poppy seeds, which showed relatively low thermal sensitivity of morphine and codeine. Indeed, our experiments indicated that the half-lives of these alkaloids at 200°C ranged from 32 to 39 min.

**FIGURE 1:**
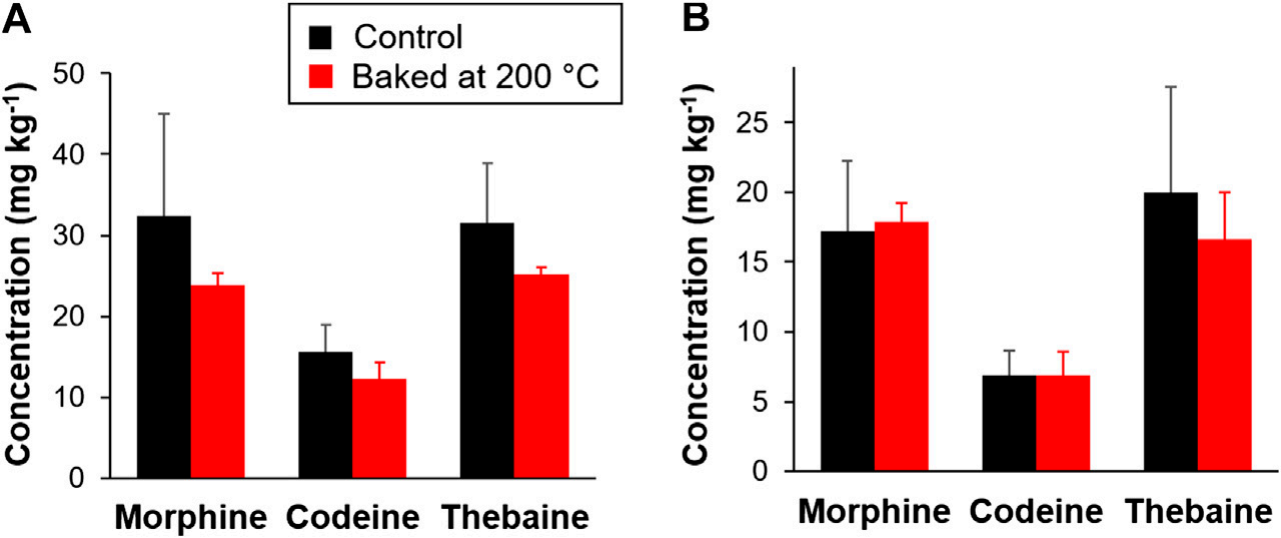
Stability of the opium alkaloids morphine, codeine, and thebaine in a model baked product. Poppy seed muffins were baked at 200°C for 16 min, extracted, and then analyzed using LC-MS/MS. **(A)** Muffin baked with 5 g poppy seeds incorporated in batter; **(B)** muffin baked with surface addition of 2.5 g poppy seeds. Values are shown as means ± SD (*n* = 3 independent replicates). No significant differences were detected using a two-tailed paired *t*-test (*p* > 0.05). Adapted with permission from Shetge et al. (2020). Copyright 2020 American Chemical Society.

We posit that the lack of a change in opium alkaloid levels in our experiments during baking in a 200°C oven may be because the interior of the muffin did not exceed 100°C under these conditions, and the surface of the muffin reached only ∼136°C at the end of the 16 min baking time period. At these temperatures, the thermal degradation data from our experiments predict that this baking time would not be of sufficient length to result in even a 50% reduction of morphine and codeine.

Although Carlin et al. cites previous work that suggested baking conditions may significantly reduce levels of opium alkaloids in poppy seeds (Sproll et al., 2006), we have previously noted that such experiments were confounded by processing steps including soaking of the poppy seeds, which has been reported to reduce opium alkaloids on the surface of poppy seeds (Shetge et al., 2020). Also, analysis of opium alkaloids by mass spectrometry can be influenced by sample matrix (López et al., 2018), but it is unclear whether the referenced baking experiments used matrix-matched standards to control for such effects.

We believe that it is important to continue to pursue research on the topic of alkaloid degradation on or in poppy seeds. As such, we acknowledge that Carlin et al. has added interesting and insightful contributions through their present work. Still, as researchers we believe that it is critical to note where the data do not support conclusions made by Carlin et al. in order to help drive this field forward. Further, as published data on reduction of opium alkaloids in poppy seeds has been used by governmental bodies to provide safety and processing recommendations in this area (CONTAM Panel et al., 2018), it is imperative that carefully designed experiments be performed in order to meaningfully inform such guidance.

## Author Contributions

The idea for the manuscript was conceptualized by BR, and all authors contributed to the writing and review of the manuscript.

## Conflict of Interest

The authors declare that the research was conducted in the absence of any commercial or financial relationships that could be construed as a potential conflict of interest.
